# Spatial Prediction and Optimized Sampling Design for Sodium Concentration in Groundwater

**DOI:** 10.1371/journal.pone.0161810

**Published:** 2016-09-28

**Authors:** Erum Zahid, Ijaz Hussain, Gunter Spöck, Muhammad Faisal, Javid Shabbir, Nasser M. AbdEl-Salam, Tajammal Hussain

**Affiliations:** 1 Department of Statistics, Quaid-i-Azam University, Islamabad, Pakistan; 2 Department of Statistics, University of Klagenfurt, Klagenfurt, Austria; 3 Faculty of Health Studies, University of Bradford, BD7 1DP Bradford, United Kingdom; 4 Bradford Institute for Health Research, Bradford Teaching Hospitals NHS Foundation Trust, Bradford, United Kingdom; 5 Department of Statistics, COMSATS Institute of Information Technology, Lahore, Pakistan; 6 Arriyadh Community College, King Saud University, Arriyadh 11437, Saudi Arabia; Institute for Health & the Environment, UNITED STATES

## Abstract

Sodium is an integral part of water, and its excessive amount in drinking water causes high blood pressure and hypertension. In the present paper, spatial distribution of sodium concentration in drinking water is modeled and optimized sampling designs for selecting sampling locations is calculated for three divisions in Punjab, Pakistan. Universal kriging and Bayesian universal kriging are used to predict the sodium concentrations. Spatial simulated annealing is used to generate optimized sampling designs. Different estimation methods (i.e., maximum likelihood, restricted maximum likelihood, ordinary least squares, and weighted least squares) are used to estimate the parameters of the variogram model (i.e, exponential, Gaussian, spherical and cubic). It is concluded that Bayesian universal kriging fits better than universal kriging. It is also observed that the universal kriging predictor provides minimum mean universal kriging variance for both adding and deleting locations during sampling design.

## Introduction

Sodium is a mineral which is always present in drinking water through natural occurrences. The human body needs sodium to maintain the blood pressure and to control fluid levels. If it exceeds a threshold value (i.e., 200 mg/l), then it may change the taste of water. Moreover, it also creates severe medical problems for those who have high blood pressure. In most of the countries the level of sodium in water is less than 20 mg/l, however, in some countries it exceeds 250 mg/l.

About 1.1 billion people in the whole world are unable to access safe drinking water, and it causes a lot of deaths. Gadgil [1] provides some guidelines and highlights the presence of required parameters in drinking water. Ferreccio et al. [2] evaluates the concentration of arsenic in drinking water which was about 860 mg/l during 1958 to 1970 in northern cities of Chile, however, later on it has been reduced to 40mg/l. They cross-examine two types of patients, smokers and non-smokers, and conclude that consumption of arsenic through water is the major cause of lung cancer. Gundogdu and Guney [3] use various kriging techniques to study the water levels by using spherical, tetraspherical, pentaspherical, exponential, Gaussian, rational quadratic, hole effect, K-bessel, J-bessel and stable variogram models. They conclude that universal kriging is better than ordinary kriging for interpolating water levels. Neuman et al. [4] described and implemented Bayesian model averaging, and maximum likelihood version of Bayesian model averaging which does not require any prior knowledge about parameters. It updates posterior probabilities as well as model parameters on the basis of new data, and is consistent with modern statistical methods of hydrologic model calibration. Mehrjardi et al. [5] show that co-kriging and kriging methods are better than inverse distance weighting techniques for predicting the spatial distribution of some characteristics of groundwater quality. Nas [6] concludes that ordinary kriging provides accurate patterns of groundwater quality parameters in Konya, Turkey. Sarukkalige [7] also uses kriging techniques for the analysis of the quality of groundwater in Western Australia. Andrade and Stigter [8] model the spatio-temporal variation of arsenic concentration in groundwater by using geostatistical and multivariate methods. They show that the concentration of arsenic has strong correlation with rice culture and that indicator kriging provides appropriate maps of arsenic concentrations.

Dhar and Datta [9] developed methodology based on inverse distance weighting method to reduce redundancy in monitoring network. Siri et al. [10] establish a sampling scheme for generating samples simultaneously. They prove that GPS units and a pseudo-sampling frame are more effective than old sampling methods. Brus and Heuvelink [11] validate that universal kriging performs better than ordinary kriging in terms of smaller mean universal kriging variance (MUKV) for spatial sampling design. Optimal spatial sampling design is a core issue in environmental studies when exploring low cost and greater efficiency samples. The sampling scheme can be optimized through prior knowledge and sampling constraints. Van Groenigen and Stein [12] conclude that Spatial Simulated Annealing (SSA) is better than classical methods for optimizing sampling designs. SSA is useful for those studies that have several sampling constraints. Zhu and Stein [13] study spatial sampling design for the estimation of covariance parameters by the maximum likelihood method.

In present paper, spatial behavior of sodium in drinking water is determined and optimized sampling patterns by both, the optimal deletion and subsequent addition of locations is generated for three divisions of Punjab, Pakistan. Several variogram models are used for modeling the spatial dependence existing in the data. Maximum likelihood (ML), restricted maximum likelihood (REML), ordinary least square (OLS), and weighted least square (WLS) are used to estimate the parameters of the variogram. Universal kriging and Bayesian kriging with varying trend are used for the prediction of sodium concentration at unobserved locations. Moreover, spatial simulated annealing optimized sampling patterns are generated.

## Materials and Methods

### Study Area

Three divisions of Punjab (Bahawalpur, Dera Ghazi Khan and Multan) are investigated for study purpose. The Bahawalpur division is a second-order administrative division that includes the districts Bahawalnagar, Bahawalpur and Rahimyar Khan. It is located at an elevation of 92 meters above sea level. Its latitude is 28°30′0” North and longitude is 71°30′0” East in Degrees, Minutes and Seconds (DMS) or 28.5 and 71.5 in decimal degrees. The Dera Ghazi Khan division has four districts (Dera Ghazi Khan, Layyah, Muzaffargarh and Rajanpur). Geographical coordinates of Dera Ghazi Khan are 30°3′22” North, 70°38′4” East. Multan division has also four districts (Khanewal, Lodhran, Multan and Vehari). Multan district is spread over an area of 3721 square km. Its latitude is 30°12′54” North and longitude is 71°35′27” East.

**Fig 1:**
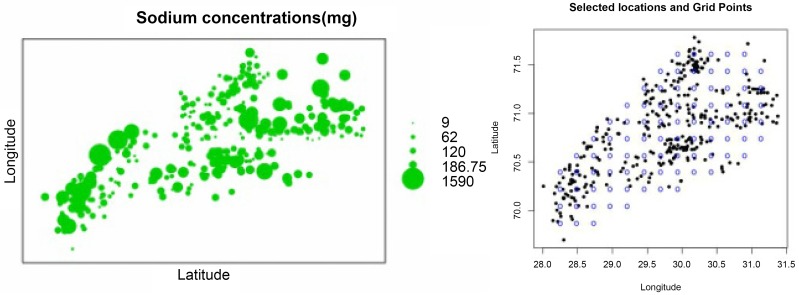
Level of sodium concentration (mg/L) at observed locations in three divisions of Punjab, Pakistan (left), and observed locations and gridded locations (right).

The Pakistan Council of Research in Water Resources (PCRWR) collected and analyzed two samples from each union council (sub-sub-sub district). The water samples were collected from easily and frequently available sources such as, hand pump, tab-water, tube-well, and water supply because it depends on inhabitants of the region. According to the PCRWR survey (2008) the samples of 370 selected sites are shown in left panel of Fig 1 and the gridded unsampled locations are presented in right panel of Fig 1. The samples of water were collected in 500 ml polystyrene bottles from the two selected sites of each union council according to a standardized method. The details about selection of samples laboratory analysis is provided in [14]. Various properties of the selected samples were analyzed according to the 2540C APHA (1992) standard.

### Spatial Interpolation Methods

Kriging is extensively used in spatial analysis and its main objective is to predict the value at an unobserved location by calculating the weighted average of samples at observed locations, see Matheron [15].

#### Universal kriging

Ordinary kriging assumes that the mean is constant in the study region, however, in universal kriging the mean is a function of the coordinates (trend). The trend can be modeled through linear or polynomial functions. Let *Y* = (*Y*_1_,……,*Y*_*n*_)^*T*^ be a vector of response variables measured at observed locations *x*_1_, *x*_2_, …,*x*_*n*_, and let its distribution be as follows:

$$Y∼N\left(\mu ,\Sigma_{Y}\right),$$ (1)

where *μ* = (*μ*(*x*_1_), *μ*(*x*_2_), …,*μ*(*x*_*n*_))^*T*^, and

$$\mu \left(x_{i}\right)=\sum_{k=1}^{p}\beta_{k}f_{k}\left(x_{i}\right),\;\text{for}\;i=1,2,...,n.$$ (2)

Here *β*_*k*_, *k* = 1, 2, …, *p*, are unknown regression coefficients and the *f*_*k*_(*x*) are known functions of the spatial coordinates *x* to ℜ^1^; *p* is the number of functions that are used to model the trend component. The covariance matrix Σ_*Y*_ is defined as

$$\Sigma_{Y}=\sigma^{2}\left(\left(1-\tau^{2}\right)R_{\alpha }+\tau^{2}I\right),$$ (3)

where *R*_*α*_ is a correlation matrix generated from one of the well known positive definite correlation function models, i.e. Gaussian, exponential or spheric. Σ_*Y*_ depends on a vector-valued parameter *θ* = (*σ*^2^ = total sill, *α* = range, *τ*^2^ = relative nugget). Equivalently one may define also the matrix of semivariogram values

$$\Gamma_{Y}=\sigma^{2}1-\Sigma_{Y},$$

where **1** is the *n*x*n*-matrix of ones. The covariance or semivariogram parameters *θ* are estimated using estimation methods like ML, restricted-ML or empirical semivariogram estimation and weighted-least-squares-fitting. The universal kriging predictor at an unsampled location *x*_0_ is a linear predictor $\hat{Y}(x_{0})=\sum_{i=1}^{n}\lambda_{i}Y_{i}$. Minimizing the mean squared error of prediction subject to the condition of unbiasedness results in the following system of simultaneous equations for the weighting vector *λ* = (*λ*_1_, *λ*_2_, …*λ*_*n*_)^*T*^:

$$\left[\begin{array}{ccccccc} \gamma_{11} & \gamma_{12} & \cdots & \gamma_{1n} & f_{1}^{1} & \cdots & f_{p}^{1}\\ \gamma_{21} & \gamma_{22} & \cdots & \gamma_{2n} & f_{1}^{2} & \cdots & f_{p}^{2}\\ \vdots & \vdots & \cdots & \vdots & \vdots & \cdots & \vdots \\ \gamma_{n1} & \gamma_{n2} & \cdots & \gamma_{nn} & f_{1}^{n} & \cdots & f_{p}^{n}\\ f_{1}^{1} & f_{1}^{2} & \cdots & f_{1}^{n} & 0 & \cdots & 0\\ \vdots & \vdots & \cdots & \vdots & \vdots & \cdots & \vdots \\ f_{p}^{1} & f_{p}^{2} & \cdots & f_{p}^{n} & 0 & \cdots & 0 \end{array}\right]\left[\begin{array}{c} \lambda_{1}\\ \lambda_{2}\\ \vdots \\ \lambda_{n}\\ l_{1}\\ \vdots \\ l_{p} \end{array}\right]=\left[\begin{array}{c} \gamma_{01}\\ \gamma_{02}\\ \vdots \\ \gamma_{0n}\\ f_{1}^{0}\\ \vdots \\ f_{p}^{0} \end{array}\right]$$ (4)

where *l*_*k*_, *k* = 1, 2, …, *p* are the Lagrange multipliers for the conditions of unbiasedness, *γ*_*ij*_ are the semivariogram values between locations *x*_*i*_ and *x*_*j*_, *i*, *j* = 0, 1, 2,…, *n* and $f_{k}^{i}=f_{k}(x_{i})$, *i* = 0, 1, 2,…, *n*, *k* = 1, 2, …, *p*. The weights vector *λ* and Lagrange multiplier *l* are calculated by solving the above system of equations and are utilized also for calculating the universal kriging variance:

$$\sigma_{uk}^{2}=\left\{\sum_{i=1}^{n}\lambda_{i}\gamma_{i0}\right\}+\left\{\sum_{k=1}^{p}l_{k}f_{k}\left(x_{0}\right)\right\}.$$ (5)

#### Bayesian Kriging

Bayesian kriging makes use of the Bayes theorem which involves the likelihood function *l* (*θ*;*Y*) and the prior distribution *π* (*θ*) of the respective parameters *θ* = (total sill = *σ*^2^,range = *α*,relative nugget = *τ*^2^,trend = *β*), see Omre [16]. The posterior distribution of the parameter vector *θ* can be expressed as:

$$\pi \left(\theta |Y\right)=\frac{l\left(\theta ;Y\right).\pi \left(\theta \right)}{\int l\left(\theta ;Y\right).\pi \left(\theta \right)d\theta }$$ (6)

All model parameters are considered uncertain, and the prior distribution of parameters is given as follows:

$$\pi \left(\beta ,\sigma^{2},\tau^{2},\alpha \right)=\pi \left(\beta |,\sigma^{2},\tau^{2},\alpha \right)\pi \left(\sigma^{2}|\tau^{2},\alpha \right)\pi \left(\tau^{2},\alpha \right),$$ (7)

whereas noninformative prior for *β* is used i.e *π*(*β*|, *σ*^2^, *τ*^2^, *α*) ∝ 1. Now, using the above prior distribution of parameters, the posterior distribution is obtained as

$$\left[\beta ,\sigma^{2},\tau^{2},\alpha |Y\right]=\left[\beta |\sigma^{2},\tau^{2},\alpha ,Y\right]\left[\sigma^{2}|\tau^{2},\alpha ,Y\right]\left[\tau^{2},\alpha |Y\right],$$ (8)

where [*β*|*σ*^2^, *τ*^2^, *α*, *Y*] is Gaussian with mean the generalized least squares estimate of *β* and covariance matrix the corresponding generalized least squares covariance matrix. The posterior density for *τ*^2^ and *α* is given as

$$\pi \left(\tau^{2},\alpha |Y\right)\propto \pi \left(\tau^{2},\alpha \right)|F^{T}R_{\tau^{2},\alpha }^{-1}{F|}^{\frac{1}{2}}{\left|R_{\tau^{2},\alpha }\right|}^{-\frac{1}{2}}{S^{2}}^{\frac{n-p}{2}},$$ (9)

where **F** is the design matrix of regression functions, **R**_*τ*^2^,*α*_ is the correlation matrix between the data and

$$S^{2}=\frac{1}{n-p}{\left(Y-F\hat{\beta }\right)}^{T}R_{\tau^{2},\alpha }^{-1}\left(Y-F\hat{\beta }\right),$$ (10)

with $\hat{\beta }$ the generalized least squares estimate of *β*. For the case that a noninformative prior *π*(*σ*^2^|*τ*^2^, *α*) ∝ 1/*σ*^2^ is used, the posterior of *σ*^2^ is given as scaled inverse chi-square distribution:

$$\sigma^{2}|\tau^{2},\alpha ,Y∼\chi^{-2}\left(n-p,S^{2}\right),$$ (11)

which is equivalent to

$$\frac{\left(n-p\right)S^{2}}{\sigma^{2}}|\tau^{2},\alpha ,Y∼\chi^{2}\left(n-p\right),$$ (12)

To predict values at unsampled locations the predictive distribution is used. Proposed by Diggle and Lophaven [17] and described in Diggle and Ribeiro [18] the predictive distribution for the unobserved signal process *Y*_0_ = *Y*(*x*_0_) at location *x*_0_ is specified as:

$$\left[Y_{0}|Y\right]=\;\int \int \int \;\left[Y_{0}|\sigma^{2},\alpha ,\tau^{2},Y\right]\left[\sigma^{2},\alpha ,\tau^{2}|Y\right]{d\sigma }^{2}d\alpha {d\tau }^{2}$$ (13)

Diggle and Ribeiro [18] use a discrete prior for the covariance parameters. This way the posterior becomes also discrete and parameters can be simulated easily from the posterior by means of multinomial sampling. Sampling from the predictive distribution is performed by first sampling a parameter vector from the discrete posterior and then with the parameter vector fixed sampling from [*Y*_0_|*σ*^2^, *α*, *τ*^2^, *Y*], which is a Gaussian distribution with mean, the universal kriging predictor, and variance, the universal kriging variance. If no discrete prior *π*(*τ*^2^, *α*) is used but a continuous one then one has to use MCMC methods to sample from the posterior *π*(*τ*^2^, *α*|*Y*).

### Spatial Sampling Design

Spatial sampling is a process in which a number of samples are used to evaluate the content of a larger geographical region. Every point in a sample exhibits information about the variable of interest at an unsampled spatial location. The sampling process has many advantages over the complete enumeration; for example, low cost, greater speed and higher scope, see Cochran [19]. The major objective of spatial sampling is to get the desired results with high precision at low cost. This can be achieved by allocating samples to locations based on minimizing an objective function, i.e., the mean universal kriging variance, see Wang et al. [20].

#### Spatial Simulated Annealing

Here, SSA is used for the optimization of the sampling design, see Van Groenigen and Stein [12]. In SSA locations are candidate measurements that are either removed or added iteratively and are optimized by minimizing the Mean Universal Kriging Variance (MUKV). The MUKV is an average of all kriging variances over a fine square grid of locations in the study region i.e.

$$MUKV=\frac{\sum \sigma_{i}^{2}}{n}$$

where $\sigma_{i}^{2}$ is the variance of universal kriging. If the trend is constant, the algorithm uses the ordinary kriging variance. As the trend varies, it uses the universal kriging variance. The SSA-algorithm used here is just standard simulated annealing algorithm as described i.e. in Kirkpatrick [21] but adapted to the spatial context by following Brus and Heuvelink [11]. In SSA two different design tasks can be accomplished by: i.) Adding *n* new sample locations to an existing monitoring-network of *m* locations. ii.) Selecting *n* < *m* samples from an existing network of *m* locations.

SSA can be described as; i): The algorithm is initialized by randomly selecting *n* design-locations from the spatial region and by calculating the MUKV based on these random locations and maybe other available *m* given locations. Let’s denote this MUKV as *MUKV*_0_. As the next step these *n* previously selected locations are randomly perturbed. Again the MUKV is calculated for *n* new locations i.e *MUKV*_1_. If *MUKV*_1_ is smaller than *MUKV*_0_ then it can be assumed that there is an improvement with last design and is stored for memorizing. If Δ_*MUKV*_ = *MUKV*_1_−*MUKV*_0_ > 0 then the previous design is accepted only with a certain probability i.e.

$$p=exp(-\frac{\Delta_{MUKV}}{T})$$ (14)

*T* is called temperature and at the beginning of the algorithm can be large. The larger *T* the higher is the probability that a worsening design will be accepted. The algorithm now starts to iterate: Every current design is randomly perturbed as described above; its MUKV is calculated and compared to the *MUKV* of the so far best design. Improvements are always accepted and stored and worsenings are accepted with the above probability, where *MUKV*_0_ takes over the role of the *MUKV* of the so far best design. Actually, the probable acceptance of worsenings prevents the algorithm from being trapped in local minima of the MUKV. Further accuracy can be achieved by accounting uncertainty of variogram parameters and kriging predictors.

### Cross Validation

Cross validation statistics are used to compare the performance of different fitted models. In the present study leave-one-out cross-validation is used for selecting appropriate variogram models, parameter estimation methods and kriging predictors. The Root Mean Squared Prediction Error(RMSPE) is used as performance measure:

$$\text{RMSPE}=\sqrt{\frac{\sum_{i=1}^{n}(\hat{Y}(x_{i})-Y(x_{i}){)}^{2}}{n}}$$

In the above statistic $\hat{Y}(x_{i})$ are the cross-validated, predicted values from a specific model and *Y*(*x*_*i*_) are the observed values of the data. Finally, the method with minimum RMSPE is used for predicting the response variable at unobserved locations.

## Results and Discussion

### Exploratory Spatial Data Analysis

**Fig 2:**
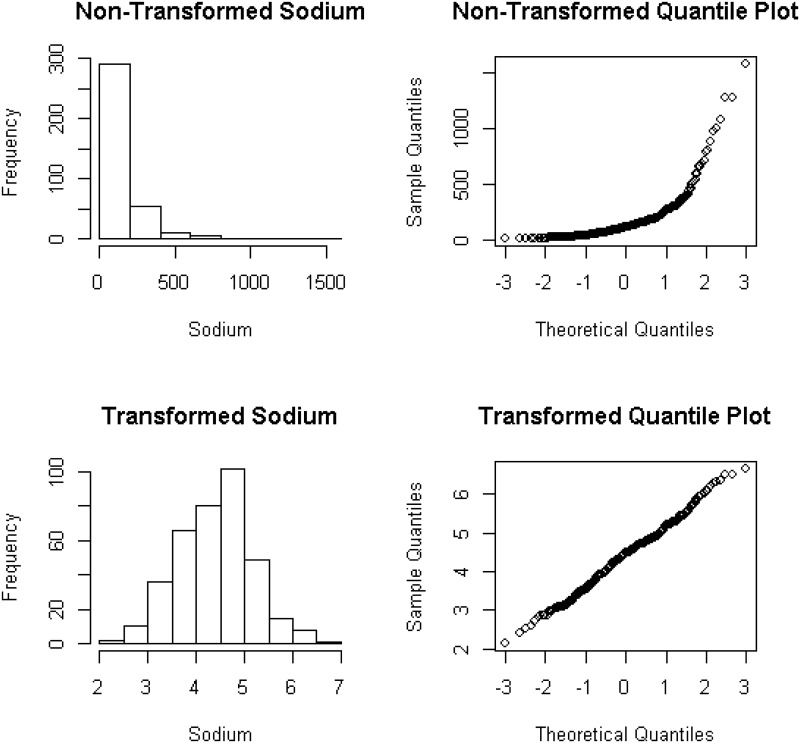
Distributions of non-transformed and transformed response variable with their respective quantile plots.

Exploratory spatial data analysis is performed on the sodium concentration data by using the geoR package of Ribeiro and Diggle [22] and R software Team [23]. This analysis is carried out to explore the spatial auto-correlation and the assumption of normality which is necessary for most of the kriging methods. It is observed that the sodium concentration data violate the assumption of normality. The Box-Cox transformation with parameter *λ* = −0.028 is used to normalize the data, see Fig 2. The transformed data are used for modeling the spatial distribution of sodium concentrations and for selecting optimal sampling designs.

### Results for Universal Kriging

**Fig 3:**
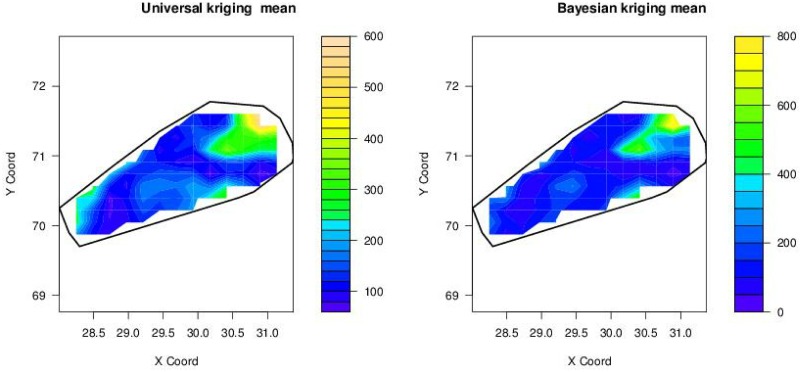
Maps of predicted sodium concentrations (mg/L) by using universal kriging (left) and Bayesian universal kriging (right), here X-coord = Latitude and Y-coord = Longitude.

**Fig 4:**
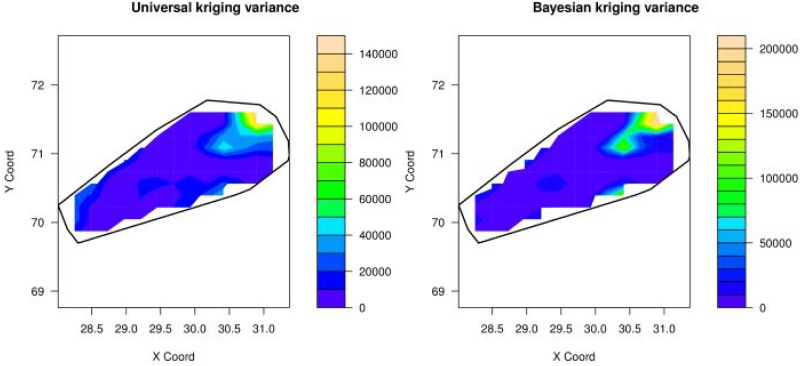
Maps of prediction variance by using universal kriging (left) and Bayesian universal kriging (right),here X-coord = Latitude and Y-coord = Longitude.

From exploratory analysis it is observed that there exists spatial dependence between sodium concentrations and coordinates, see left panel of Fig 1. Universal kriging with linear trend can take into account this spatial dependence. Since modeling the variogram is essential for prediction by kriging, various variogram models are fitted through different estimation methods (results of RMSPE given in Table 1). Table 1 shows that REML provides minimum RMSPE for covariance (see column 2-5 of Table 1). REML and universal kriging provide minimum RMSPE as REML has the advantage of removing the trend during variogram estimation. From the RMSPE presented in Table 1 it can be concluded that universal kriging with a spherical variogram model fits well under REML. The estimated parameters of the best fitting model are given in Table 2. Thus, the spherical model is used for the prediction of sodium concentrations in drinking water. The contour plots of predicted values and prediction variances are given in left panels of Figs 3 and 4, respectively. Left panel of Fig 3 shows that the concentration of sodium is highly exceeding the limit, which is 200*mg*/*l* in the region between (28.3° − 28.5° latitude and 70° − 70.5° longitude), (30.2° − 30.4° latitude and 70.2° − 70.4° longitude) and (30.1° − 31.1° latitude and 70.9° − 71.5° longitude). Left panel of Fig 4 shows that in most of the area the variance of prediction is small except in the region between (30.2° − 30.7° latitude and 70.9° − 71.2° longitude).

**Table 1:** RMSPE of spatial covariance models subject to different methods of estimation (ML, REML, OLS, and WLS) and universal kriging.

Models	ML	REML	OLS	WLS
Universal kriging				
Expon.	178.227	177.208	179.242	187.428
Gaussian	179.244	178.612	180.022	182.425
Spherical	177.998	176.603	181.518	181.578
Cubic	178.731	178.142	179.551	179.993

**Table 2:** Parameters of the variogram model subject to REML estimation and universal kriging.

Spherical Model			
Methods	Sill	Range	Nugget
Universal kriging	0.7078	1.1709	0.43

### Results for Bayesian Kriging

Bayesian kriging with linear trend is used with spherical and exponential variogram models. The prior distributions are specified as follows: for the mean parameter *β* a uniform prior is used, i.e *p*(*β*) ∝ 1; for the range parameter *α* a uniform prior is used, too, i.e *p*(*α*) ∝ 1; the prior for *σ*^2^ (total sill) is scaled inverse chi-square with 367 degrees of freedom; and for the relative nugget parameter *τ*^2^ a uniform prior is used, too. The exponential and spherical covariance models are investigated. Leave-one-out cross-validation is used to estimate the RMSPE for each model. The RMSPE presented in Table 3 shows that the spherical model fits well (minimum RMSPE) for Bayesian kriging with linear trend. For predicting the sodium concentration at unsampled locations the posterior predictive distribution is used to draw contour maps. Right panel of Fig 3, showing the posterior predictive means, indicates that sodium concentrations are high in the regions (28.3° − 28.6° latitude and 69.9° − 70.5° longitude), (29° − 29.7° latitude and 70.1° − 70.7° longitude), and concentration of sodium is a serious issue in the regions (30.1° − 31.1° latitude and 70.9° − 71.5° longitude) and (30.3° − 30.5° latitude and 70.2° −70.4° longitude). From right panel of Fig 4 it can be observed that in most of the area the posterior predictive variance is similar except in the region (30.5° − 31.0° latitude and 71.4° − 71.6° longitude), where it is extremely high.

**Table 3:** RMSPE of spatial covariance models subject to Bayesian universal kriging.

RMSPE		
Methods	Exponential	Spherical
Bayesian univ. kriging	177.195	175.601

### Comparison of Spatial Interpolation Methods

Root mean squared errors of prediction of the spatial interpolation methods are given in Table 4. The RMSPE of Bayesian universal kriging is less than the RMSPE of universal kriging. It can be conclude that Bayesian universal kriging performs better than universal kriging. This is due to the use of additional information, data information as well as prior information, and more statistical modeling flexibility of Bayesian universal kriging.

**Table 4:** Comparison of the interpolation methods.

Methods	RMSPE
Universal kriging	176.603
Bayesian universal kriging	175.601

## Optimized Sampling Design

One of the objectives of the present paper is to obtain an optimum allocation of sampling locations to minimize cost and prediction error. Since the kriging prediction variance depends on both the sample size, the variance and covariance of the sample. Optimum allocation can be obtained only by considering all of these factors. Different variogram models are fitted as it is a pre-requisite for prediction by kriging. The variogram model providing minimum RMSPE is considered as appropriate for prediction and sampling design. For selecting the optimal sampling design optimal deletion and subsequent addition of locations are performed. The diameter was fixed by k-means i.e *k* = 6 and temperature was fixed by following Brus and Heuvelink [11].

Table 5 shows the MUKV using the observed 370 data locations for both, ordinary and universal kriging, and a spherical variogram model which comes out to be the appropriate model.

**Table 5:** MUKV vs. sample size using spherical variogram model.

Method	Sample Size	MUKV
Ordinary kriging	370	24018
Universal kriging	370	23930

### Deleting Locations using Spatial Simulated Annealing

**Fig 5:**
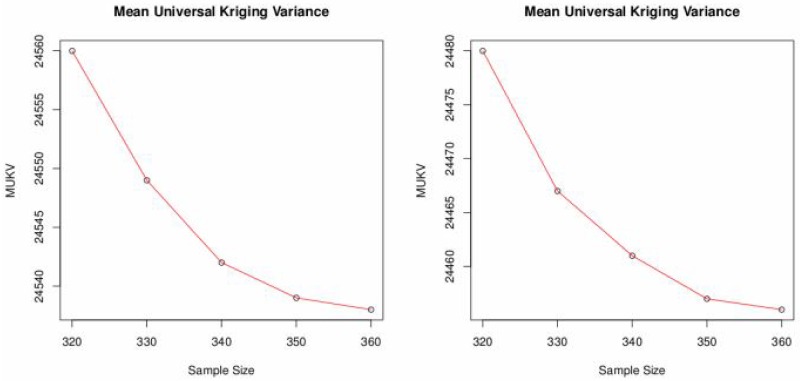
MUKV vs. sample size using ordinary and universal kriging.

**Fig 6:**
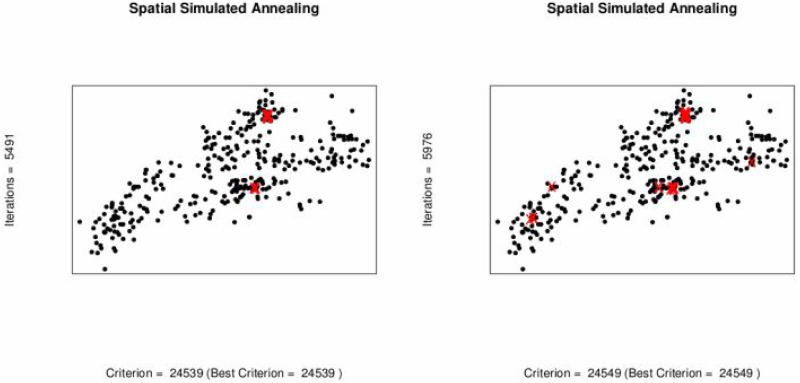
Optimal sampling design for ordinary kriging when locations are deleted,(Red).

**Fig 7:**
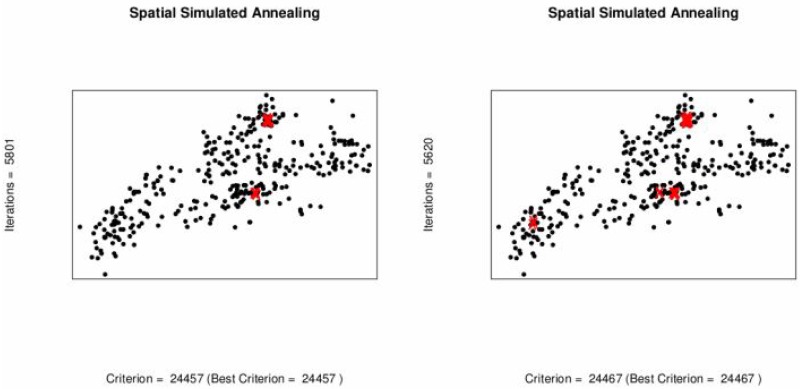
Optimal sampling design for universal kriging when locations are deleted (Red).

To minimize sampling cost one possible option is to reduce the number of sampling locations. Spatial simulated annealing (SSA) is used to optimize the effect of deleting sampling locations upon MUKV. The MUKV is calculated using ordinary and universal kriging. The MUKV increases in both kriging techniques as the number of data locations goes down, see Table 6 and Fig 5. Subsequently deleting 20 and 40 locations from the 370 locations. When deleting 50 locations from the 370 locations the MUKV using ordinary kriging is 24560 and the MUKV for universal kriging is 24480, see Tables 5 and 6. According to the MUKV criterion it can be inferred that universal kriging theoretically seems to perform better than ordinary kriging. The sampling patterns for deleting locations using ordinary kriging and universal kriging are shown in Figs 6 and 7. Algorithm stops on getting the optimum criterion i.e., MUKV. It can be observed that number of iterations performed by the algorithm are different in every sub figure. Obviously, only redundant locations in densely sampled areas are deleted, resulting in only a slight increase of the MUKV.

**Table 6:** MUKV vs. sample size using spherical variogram model for deleting locations.

Method	Sample Size	MUKV
Ordinary Kriging	370-10	24538
	370-20	24539
	370-30	24542
	370-40	24549
	370-50	24560
Universal Kriging	370-10	24456
	370-20	24457
	370-30	24461
	370-40	24467
	370-50	24480

### Adding Locations using Spatial Simulated Annealing

**Fig 8:**
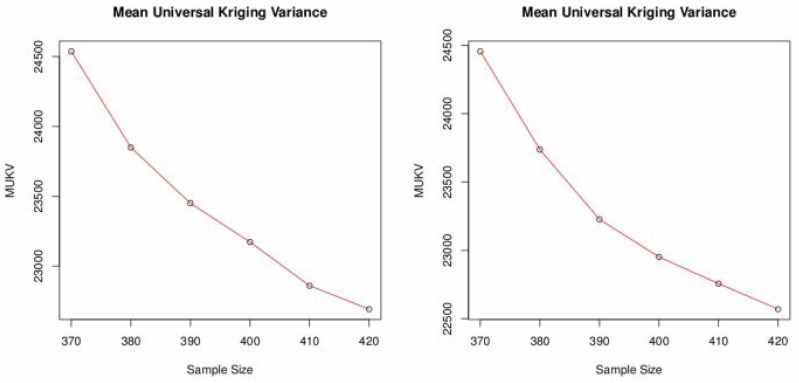
MUKV vs. sample size using ordinary and universal Kriging.

**Fig 9:**
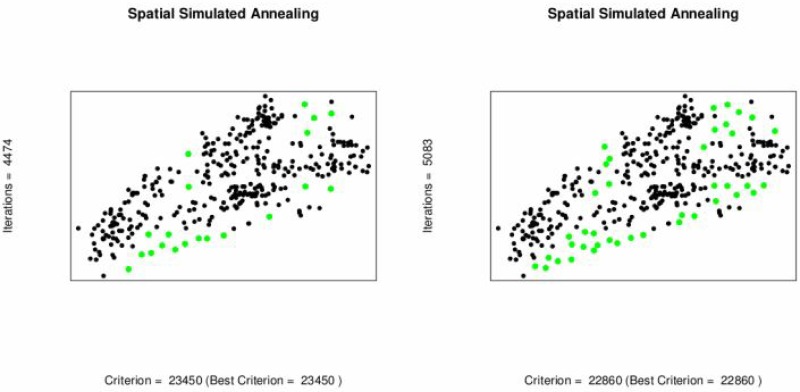
Sub-optimal 370+40 point design, ordinary kriging. Green: added locations.

**Fig 10:**
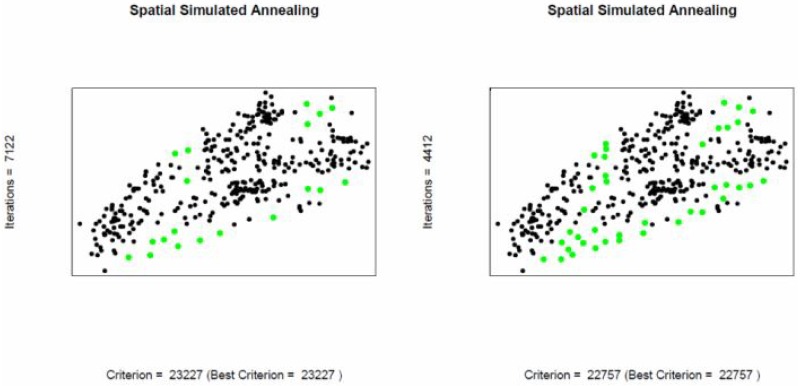
Suboptimal 370+40 point design, universal kriging. Green: added (20 &40) locations.

Like before the objective here is to find the sampling pattern by adding locations that has minimum MUKV. If the number of locations to be added are increased in the SSA-algorithm then the MUKV decreases for both, ordinary and universal kriging, see Fig 8 and Table 7. However, the mean universal kriging variance is smaller than the ordinary kriging one. The sampling patterns after adding locations using ordinary kriging and universal kriging are shown in Figs 9 and 10. Both sampling designs look quite similar and space-filling.

**Table 7:** MUKV by varying the sample size using a spherical model for adding locations.

Method	Sample Size	MUKV
Ordinary Kriging	370+10	23849
	370+20	23450
	370+30	23173
	370+40	22860
	370+50	22692
Universal Kriging	370+10	23737
	370+20	23227
	370+30	22952
	370+40	22757
	370+50	22570

## Conclusion

The present paper is focused on two objectives: (i) to model the spatial distribution of sodium concentration, and (ii) to generate optimized spatial sampling designs for three divisions of Punjab, Pakistan. Universal kriging and Bayesian kriging with varying linear trend are used for modelling the spatial distribution of sodium concentrations in drinking water. To take account of the spatial dependence in the response variable Gaussian, exponential, spherical and cubic variogram models are used. It is concluded that the spherical variogram model provides smaller mean squared error of prediction than other variogram models. Since Bayesian universal kriging has the advantage of utilizing prior information about model parameters and is statistically more flexible, it performs better than universal kriging.

**Fig 11:**
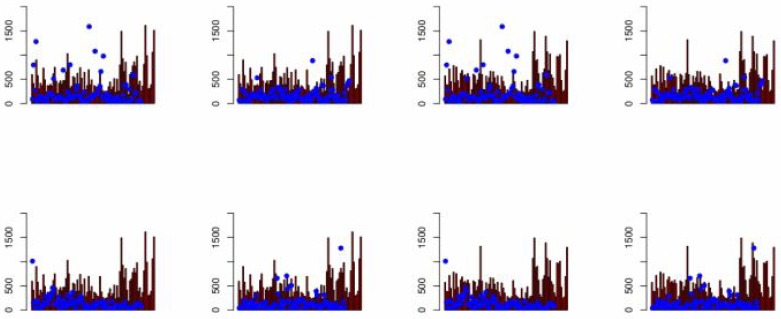
The 95% predictive interval plots and actual values of data for universal kriging (left) and Bayesian Universal Kriging (right), the dots represent to actual interval and bars represent to 95% credible intervals.

A comparison of the used kriging methods can also be based on credible intervals. The 95% credible intervals are estimated from the simulated values of sodium concentration. Plots of credible intervals as presented in Fig 11 show that Bayesian universal kriging has shorter intervals than universal kriging. In Bayesian universal kriging most of the actual data values lie within the 95% predictive intervals. Thus, Bayesian universal kriging gives more reliable predictions than universal kriging. This is also due to the fact that Bayesian universal kriging does take into account the uncertainty of the covariance model. Universal kriging does not; once the covariance model is estimated it is fixed and its uncertainty is discarded during prediction. Prediction maps of sodium concentrations are generated based on Bayesian universal and universal kriging; locations having sodium concentration above the threshold value of 200mg are identified. Prediction maps show that sodium concentrations in drinking water are increasing to the southern side of the study area.

Spatial simulated annealing is used to generate optimized spatial designs for adding and deleting locations. For optimization the MUKV is used as objective function subject to ordinary kriging or universal kriging. When deleting locations the MUKV increases; however, the increase is not much because redundant locations are deleted. If locations are added the MUKV decreases; the designs themselves have a space-filling character, when adding locations by means of SSA. Spatial simulated annealing optimize the patterns, which are generated by deleting and adding locations. Time and cost may be saved by deleting the redundant locations, and the variation can be minimized by adding locations that was unfilled. Recently, Junez-Ferreira and Herrera [24] used Kalman filter to sequentially optimize space-time monitoring points. Their suggested method can minimize the prediction error in better way as compared with simulated annealing algorithm. For further improvement in optimizing sampling design in present paper the method suggested in [24] can be used.

## Supporting Information

S1 File R-Language program used for statistical analysis, “S1 File.r”. (R)

S2 File Data used in the manuscript “S2 File.csv”. (CSV)
